# Pupil dynamics reveal preparatory processes in the generation of pro-saccades and anti-saccades in open skill sports athletes

**DOI:** 10.5114/biolsport.2026.153308

**Published:** 2025-08-05

**Authors:** Jui-Tai Chen, Yi-Hsuan Chang, Cesar Barquero, Chin-An Wang

**Affiliations:** 1 Department of Anesthesiology, School of Medicine, College of Medicine, Taipei Medical University, Taipei City, Taiwan; 2 Department of Anesthesiology, Shuang Ho Hospital, Taipei Medical University, New Taipei City, Taiwan; 3 Institute of Cognitive Neuroscience, College of Health Science and Technology, National Central University, Taoyuan City, Taiwan; 4 Eye-Tracking Laboratory, Shuang Ho Hospital, Taipei Medical University, New Taipei City, Taiwan; 5 Department of Physical Activity and Sport Science, Universidad Peruana de Ciencias Aplicadas, Lima, Peru; 6 Ph.D. Program in Medical Neuroscience, College of Medical Science and Technology, Taipei Medical University, Taipei City, Taiwan

**Keywords:** Executive function, Cognitive load, Pupil dilation, Superior colliculus, Frontal eye field, Preparatory set, Volitional control

## Abstract

This study investigated pupil dynamics to establish a physiological index of mental processes associated with executive functioning, enabling objective evaluation of cognitive load during training to improve understanding of cognitive control in sport-specific contexts. Using video-based eye-tracking, we examined pupil and saccade responses in athletes (N = 40) and non-athletes (N = 40) performing an interleaved prosaccade and anti-saccade task. In this task, participants were instructed prior to target appearance to either make a reflexive saccade toward the target (pro-saccade) or inhibit that response and generate a voluntary saccade in the opposite direction (anti-saccade). Larger pupil dilation prior to target onset was observed during anti-saccade compared to pro-saccade preparation (p < 0.001, ηp^2^ = 0.153). Athletes showed reduced pupil dilation compared to non-athletes (p < 0.05, ηp^2^ = 0.049). In addition, trials with larger pupil dilation and smaller tonic pupil sizes were associated with faster saccade reaction times. Pupil dilation also positively correlated with saccade peak velocities but showed no association with saccade endpoint accuracy. These findings suggest that athletes may engage in more efficient motor preparation, as reflected by reduced pupil dilation. Moreover, phasic pupil dilation, indexing cognitive load, and tonic pupil size, associated with arousal level, both contributed to the control of saccade dynamics during goal-directed movements. Together, these results highlight the utility of pupil size as an objective and informative index for assessing both cognitive and arousal functions in sports science research.

## INTRODUCTION

The capacity to respond adaptively to the same sensory input is a critical cognitive skill in athletes. In dynamic environments, athletes must frequently select different responses to identical cues depending on contextual demands and strategic goals. This adaptability has been linked to variations in response readiness and the intention to execute a specific action, concepts encapsulated by the term preparatory set [1, 2]. An interleaved pro- and anti-saccade task has been widely used to investigate this preparatory set [3–6], in which participants are instructed in advance, prior to the appearance of a target, to either generate a pro-saccade toward a peripheral stimulus or an anti-saccade in the opposite direction. Unlike pro-saccades, which reflexively direct the eyes toward a stimulus, anti-saccades require the suppression of this automatic response and the voluntary initiation of a saccade in the opposite direction. While various cognitive functions have been examined in sports science research [7, 8], research investigating preparatory set remains very limited.

Distinct preparatory neural activity in several cortical and subcortical regions, particularly the superior colliculus (SC) and frontal eye fields (FEF) in the prefrontal cortex, must be engaged before target appearance to enable the execution of pro- or anti-saccades according to task demands [3–6]. Specifically, greater FEF activation has been observed during the preparation of anti-saccades compared to pro-saccades [9, 10]. Moreover, this preparatory activity correlates with saccadic reaction times (SRT), with faster SRTs associated with higher levels of activation [11, 12].

Pupil size has become an increasingly accessible and effective index in cognitive neuroscience research [13], as it is modulated by a range of high-level cognitive processes [14, 15], in addition to its well-established responses to global luminance levels [16]. While the locus coeruleus (LC) is commonly implicated in high-level pupil modulations [17], the oculomotor circuit coordinated by the SC (or FEF), also plays a central role in pupil control [18, 19]. Notably, pupil dilation can be evoked through SC microstimulation, underscoring a direct link between oculomotor activity and pupil responses [20–22]. Preparatory activity mediated by the oculomotor system should therefore be reflected in pupil size. In line with this, studies have reported larger pupil dilation during anti-saccade compared to pro-saccade preparation, and a negative correlation between pupil dilation magnitude and SRTs [23–29], supporting the use of pupil dilation as an effective index for probing brain circuits underlying preparatory set. While pupil dilation has proven useful in characterizing preparatory activity, the full dynamics of pupil responses, potentially offering additional insights into neural function and circuit dynamics [30–32], have not been extensively examined in this context. Furthermore, beyond its role in regulating saccade latency, the oculomotor circuit is also involved in the control of saccade metrics such as peak velocity and endpoint accuracy [33, 34]. However, the extent to which pupil responses reflect saccade metrics remains largely unknown.

The objective of this study was to investigate preparatory set differences between athletes and non-athletes. An interleaved pro- and anti-saccade paradigm was used, with a gap period (no stimulus) inserted between fixation point disappearance and peripheral target appearance to probe preparatory processes underlying the generation of pro- or anti-saccades. This study presents a novel analysis of pupil dynamics based on data previously collected and partially reported in an earlier study [35]. Specifically, we newly examined both phasic pupil dilation and tonic pupil size, as well as their relationships with saccade metrics, to gain deeper insight into the neural mechanisms of motor preparation and arousal in athletes. We hypothesized that previously documented preparatory activity related to pro- and anti-saccade preparation would be reflected in pupil dilation, given that both the SC and FEF are causally involved in the control of pupil size [20–22]. Moreover, we expected that athletes would exhibit more efficient preparation than non-athletes, as a substantial body of research has shown that athletes tend to demonstrate cognitive advantages across a range of cognitive tasks compared to non-athletes [7, 8]. Furthermore, because the SC is also involved in the control of saccade metrics [33, 34], we hypothesized that pupil dilation would correlate with saccade metrics as well. Finally, we also explored the role of pupil dynamics in shaping saccade behavior.

## MATERIALS AND METHODS

### Participants

All experimental procedures were reviewed and approved by the Institutional Review Board of Taipei Medical University, Taiwan, and were conducted in accordance with the Declaration of Helsinki (World Medical Association, 2001). All participants had normal or corrected-to-normal vision and were naïve to the purpose of the experiment. They provided informed consent and were compensated for their participation. Eighty participants were recruited through university advertisements. Athletes from open-skill sports were selected because they often perform better in cognitive tasks compared to those from closed-skill sports sports [36–39]. The athlete group consisted of university students aged 20–30 years (20 females, 20 males; mean age ± SD: 22.4 ± 2.5 years) who were members of table tennis (N = 27, 4–20 years of experience, 9.1 ± 3.9 years), tennis (N = 11, 3–14 years of experience, 6.5 ± 3.6 years), or badminton (N = 2, 10–11 years of experience, 10.5 ± 0.7 years) teams at the university. The non-athlete group (hereafter referred to as NON) comprised university students aged 21–30 years (20 females, 20 males; mean age ± SD: 25.5 ± 2.7 years) who did not belong to any university sports team. Since no prior studies have examined pupil size using the interleaved pro- and anti-saccade task in athletes, the sample size was determined based on our previous studies involving similar pupil size measurements and trial numbers per participant [25–27]. Some analyses of this dataset have been reported previously [35], and the pupil data have not been analyzed previously and all analyses presented here are new.

### Recording and Apparatus

Participants were seated in a dark room, with their heads stabilized in a chin and forehead rest, and the only light source was the stimulus display (LCD screen). Eye position was measured with a videobased eye tracker (Eyelink-1000 plus binocular-arm, SR Research, Osgoode, ON, Canada) at a rate of 1000 Hz with binocular recording, and stimulus presentation and data acquisition were controlled by the Eyelink Experiment Builder. Stimuli were presented on an LCD monitor at a screen resolution of 1920 x 1080 pixels with a 60 Hz refresh rate, subtending a viewing angle of 43° × 24°, with the distance from the eyes to the monitor set at 80 cm. We used the method suggested by Eyelink to transfer output pupil area values recorded from the eye tracker to actual pupil size in diameter.

### Interleaved pro- and anti-saccade task

**FIG. 1:**
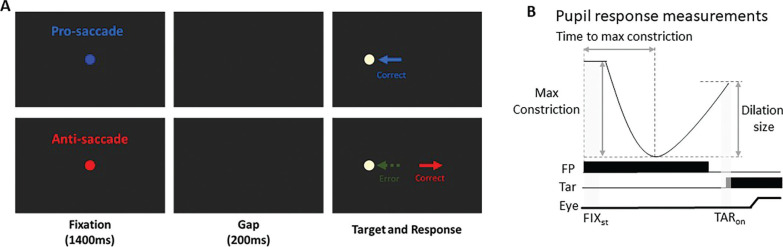
(A) Each trial started with a central colored fixation point on a black background. After 1400 ms, a blank screen was presented for 200 ms (gap) before target stimulus presentation. Participants were required to move their eyes to the stimulus in the pro-saccade condition, but move to the opposite location in the anti-saccade condition. Note that the displayed FP colors here are only for illustration of the paradigm. (B) Two selected epochs for pupil analyses: FIX_st_ (fixation start): 0 - 200 ms after fixation onset; TAR_on_ (target onset): -50 - 50 ms after target onset. FP: fixation point, Tar: target, Eye: eye position.

Following our previous study [27], each trial began with the appearance of a central fixation point (FP) (0.5° diameter, 20 cd/m^2^) on a black background (0.1 cd/m^2^) (Fig. 1). The trial condition was indicated by the fixation point (FP) color, with one color representing pro-saccades and another representing anti-saccades, and the assignment of colors to the pro- or anti-saccade conditions was counterbalanced across participants. After 1400 ms of central fixation, the FP disappeared for 200 ms (gap) before the peripheral target stimulus appeared (0.5° diameter; yellowish white dot with luminance 270 cd/m^2^) to the left or right of the FP (8° eccentricity on the horizontal axis). On pro-saccade trials, the participants were instructed to look towards the peripheral target stimulus as soon as it appeared. On anti-saccade trials, the participants were instructed to look in the opposite direction of the target stimulus (i.e., its mirror position) as soon as it appeared. Trial condition (pro- and anti-saccade) and target location (left and right) were randomly interleaved. Saccades toward either the right or left direction were combined for data analysis. The experiment comprised 180 trials.

### Data analysis

As described in detail [35], there are differences in saccade reaction times (SRT) between the two groups. The mean correct SRTs for non-athletes were 165 ± 38 ms and 230 ± 42 ms in the pro- and anti-saccade conditions, respectively, while for athletes, they were 148 ± 31 ms and 216 ± 38 ms. No significant group differences were observed in direction error rates. The mean direction error rates for non-athletes were 3.3 ± 3% and 15 ± 11% in the pro- and antisaccade conditions, respectively, while for athletes, they were 3.7 ± 4% and 18.9 ± 12%. Unlike a previous study [35], the present study focused on pupil size and its association with pro- and anti-saccade generation. SRT (or saccade latency) was defined as the time from target appearance to the initiation of the first saccade away from fixation, identified when eye velocity exceeded 30°/s. Trials were considered correct if the first saccade after target appearance was in the correct direction (toward the stimulus in the prosaccade condition or away from the stimulus in the anti-saccade condition). Direction errors occurred when the first saccade after target appearance was in the wrong direction, such as toward the stimulus on anti-saccade trials. Express saccades, characterized by short-latency, stimulus-driven responses, were defined as occurring when the proportion of anti-saccade error trials exceeded that of correct anti-saccade trials (binomial sign test, p < 0.05). This resulted in an express saccade time window of 80–140 ms after target appearance [35]. Additionally, we calculated saccade metrics including saccade amplitude (saccade size in degrees), saccade peak velocity (deg/s), and endpoint accuracy (angular deviation of the end position of the first saccade from the correct saccadic location) [35]. Trials were excluded from analysis if eye position deviated by more than 2° from the central fixation point during the required central fixation period. Additionally, outlier trials were removed if they exhibited an endpoint deviation exceeding 8° or peak velocities exceeding 800°/s, as saccades with an 8° amplitude typically have peak velocities of approximately 400°/s [40]. Applying these criteria resulted in the removal of approximately 6% of trials. For the analysis of express saccades, two non-athlete participants were excluded because they had no express saccades in the pro-saccade condition.

Pupil size data can be distorted by eye position because the measured pupil size in a video-based eye tracker depends on the angle of the eyeball. To ensure accurate pupil size measurements, the selected epochs for pupil analysis were restricted to periods when eye position was at the center of the screen, either during the central fixation period or before saccade initiation. Following the recommended procedure [41], we used MATLAB scripts for pupil data preprocessing. Invalid pupil values, such as those caused by blinks, were identified, and linear interpolation was applied using pre- and post-invalid pupil values to replace missing data. Pupil metrics were analyzed based on established methodologies [30–32], and five pupil measures were reported (Fig. 1B). First, tonic pupil size (pre-stimulus) at baseline (absolute baseline pupil diameter) was determined by averaging pupil size over the first 0–200 ms after fixation onset (FIXst: 0–200 ms after fixation onset). This baseline value was subtracted from the original pupil values to analyze four additional indices related to phasic pupil responses [32]. We calculated the maximum constriction magnitude and the time of maximum constriction (i.e., the time at which pupil size reached its smallest value, referred to as “time to max constriction”). Additionally, pupil size was measured during the target epoch (TARon: -50 to 50 ms relative to target onset). Importantly, previous research has demonstrated that a later dilation component of the pupil response is associated with saccade preparation [23–27]. To quantify this, we calculated pupil dilation by subtracting the maximum constriction pupil size from the pupil size during the target epoch (i.e., TARon pupil size minus max constriction magnitude). A mixed ANOVA (2 x 2 ANOVA: between-subjects factor: Athletes/non-athletes × within-subjects factor: experimental conditions) was performed for statistical analysis. For some analyses, independent student *t* tests (two-sided) were conducted. To examine the relationship between pupil dynamics and saccade behavior, trial-by-trial correlational analysis was conducted within each participant and subsequently tested at the group level. Most of these correlational analyses were exploratory, and thus their interpretations are offered with appropriate caution and without causal inference. However, the specific relationship between pupil dilation and SRT was inspired by existing literature reporting negative correlations between these measures [26, 27]. Therefore, for this analysis, a one-sided t-test was used to test the directional hypothesis that larger pupil dilation would be associated with faster SRTs. A conventional alpha level of p < .05 was used to determine statistical significance for all analyses. Effect sizes (partial eta squared or Cohen’s d), where appropriate, are also reported. Statistical tests were performed using JASP Team (2019) and MATLAB (The MathWorks Inc., Natick, MA, USA).

## RESULTS

### Pupil dynamics before target appearance

**FIG. 2:**
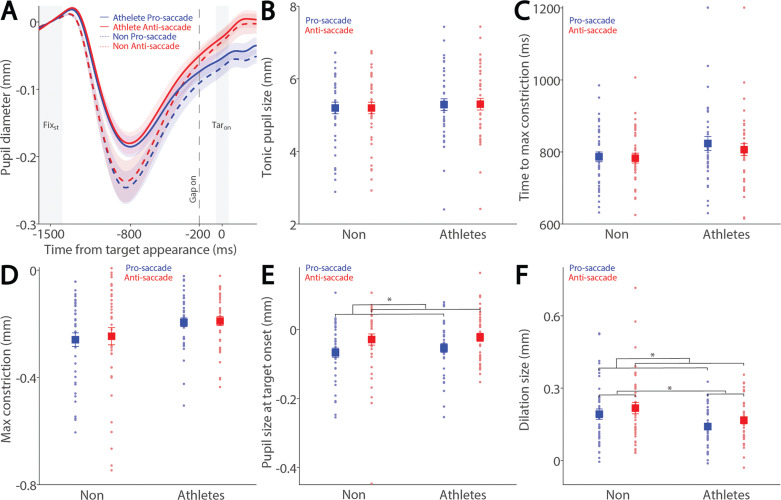
Pupil size during instructed fixation between athletes and non-athletes. (A) Pupil dynamics (baseline-corrected to FIX_st_) between the pro- and anti-saccade conditions in athletes (N = 40) and non-athletes (N = 40). (B) Tonic pupil size in the FIX_st_ epoch is shown for different conditions between athletes and non-athletes. (C) Time to max constriction is shown for different conditions between athletes and non-athletes. (D) Max constriction magnitude is shown for different conditions between athletes and non-athletes. (E) Pupil size in the TAR_on_ epoch is shown for different conditions between athletes and non-athletes. (F) Dilation size is shown for different conditions between athletes and non-athletes. The shaded colored regions surrounding pupil dynamics curves represent the ± standard error range (across participants) for different conditions. The color-filled squares and error bars represent mean value ± standard error (across participants) for each condition, and the small circles represent mean value for each subject. Color dots represent each data point. The grey area represents the epoch selected for analyses. * indicates differences are statistically significant. Non: non-athletes.

To examine how saccade preparation (pro- and anti-saccades) modulates pupil dynamics in athletes and non-athletes, we analyzed pupil dynamics during the instructed fixation period before target appearance in correct trials. Figure 2A shows pupil size baselinecorrected to the diameter at fixation onset (FIX_st_), with an initial pupil constriction followed by dilation during the instructed fixation period in both conditions, revealing differences between the two groups. Tonic pupil size during the FIX_st_ epoch, an index of tonic LC activity [17], was similar between groups (Fig. 2B: F_(1, 78)_ = 0.195, p = 0.660, ηp^2^ = 0.002), with no significant main or interaction effects (p > 0.2). In contrast, group differences were observed in phasic pupil responses. The initial pupil constriction was primarily driven by changes in global luminance following the presentation of the luminant FP (commonly referred to as the pupil light reflex). Task-dependent pupil size differences between the pro- and antisaccade conditions began to emerge at approximately 800 ms after instructed fixation onset, with larger pupil sizes observed in antisaccade trials compared to pro-saccade trials in both groups. More specifically, time to maximum constriction was longer in non-athletes compared to athletes, though this difference was not statistically significant (Fig. 2C: group: F_(1, 78)_ = 2.160, p = 0.146, ηp^2^ = 0.027), with no significant main or interaction effects (p > 0.4). Similar results were observed for max constriction magnitude (Fig. 2D: group: F_(1, 78)_ = 3.753, p = 0.056, ηp^2^ = 0.046; task: F_(1, 78)_ = 0.800, p = 0.374, ηp^2^ = 0.010; interaction: F_(1, 78)_ = 0.160, p = 0.690, ηp^2^ = 0.002). Larger pupil sizes were observed in the antisaccade condition compared to the pro-saccade condition (Fig. 2E: task: F_(1, 78)_ = 14.138, p < 0.001, ηp^2^ = 0.153), with no other significant effects (p > 0.5). More importantly, we focused on the dilation component, as it is linked to task preparation and executive functioning [23–27]. As shown in Figure 2F, smaller pupil dilation was observed in athletes compared to non-athletes (F_(1, 78)_ = 3.977, p < 0.050, ηp^2^ = 0.049). Additionally, consistent with previous research [23–27, 29], larger pupil dilation was observed in the anti-saccade condition compared to the pro-saccade condition (F_(1, 78)_ = 14.058, p < 0.001, ηp^2^ = 0.153), with no significant interaction effect (p > 0.9). Furthermore, because athletes were younger on average, age was included as a covariate in a follow-up analysis (ANCOVA) to confirm group differences. While the group effect remained comparable in magnitude after controlling for age (F_(1, 77)_ = 3.963, p = 0.050, ηp^2^ = 0.049), it no longer reached conventional statistical significance. The effect of age itself was not significant (F_(1, 77)_ = 0.328, p = 0.569, ηp^2^ = 0.004). Furthermore, investigating pupil dynamics between correct trials and directionerror trials in the anti-saccade condition revealed no reliable differences (Supplementary Fig. 1). Together, these results demonstrate group differences in task preparation, as measured by pupil dilation size.

### Larger pupil dilation in preparation for faster saccades

**FIG. 3:**
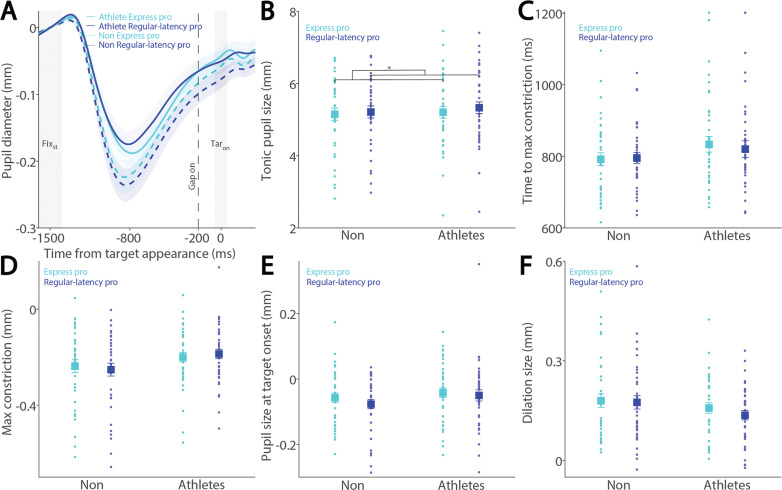
Pupil size for trials with faster and slower pro-saccades between athletes and non-athletes. (A) Pupil dynamics between the pro- and anti-saccade conditions in athletes and non-athletes. (B) Tonic pupil size in the FIX_st_ epoch is shown for different conditions between athletes and non-athletes. (C) Time to max constriction is shown for different conditions between athletes and non-athletes. (D) Max constriction magnitude is shown for different conditions between athletes and non-athletes. (E) Pupil size in the TAR_on_ epoch is shown for different conditions between athletes and non-athletes. (F) Dilation size is shown for different conditions between athletes and non-athletes. The shaded colored regions surrounding pupil dynamics curves represent the ± standard error range (across participants) for different conditions. The color-filled squares and error bars represent mean value ± standard error (across participants) for each condition, and the small circles represent mean value for each subject. Color dots represent each data point. The grey area represents the epoch selected for analyses. * indicates differences are statistically significant. Non: non-athletes.

Preparatory activity during saccade planning correlates with SRTs on both pro- and anti-saccade trials. This preparatory activity is also reflected in pupil dilation, as pupil dilation magnitude has been shown to correlate with SRTs [26, 27]. To investigate the relationship between pupil metrics and SRTs, we systematically examined their correlation in both athletes and non-athletes. We first separated express saccades from regular-latency saccades in correct pro-saccade trials, defining express saccades as those with SRTs between 80 and 140 ms (see Methods). Figure 3A shows the dynamics of baselinecorrected pupil size before target appearance in correct pro-saccade trials. Smaller tonic pupil sizes were observed in express saccade trials compared to regular-latency saccade trials (Fig. 3B, F_(1, 78)_ = 13.881, p < 0.001, ηp^2^ = 0.154), with no other significant effects (p > 0.2). No significant effects of saccade type, group, or interaction were found for time to maximum constriction (Fig. 3C, p > 0.1), max constriction magnitude (Fig. 3D, p > 0.1), or pupil size at target onset (Fig. 3E, p > 0.1). Although larger pupil dilation was observed in express saccade trials compared to regular-latency saccade trials, this difference did not reach significance (Fig. 3F, F_(1, 78)_ = 2.925, p = 0.091, ηp^2^ = 0.037), with no other significant effects (p > 0.1).

**FIG. 4:**
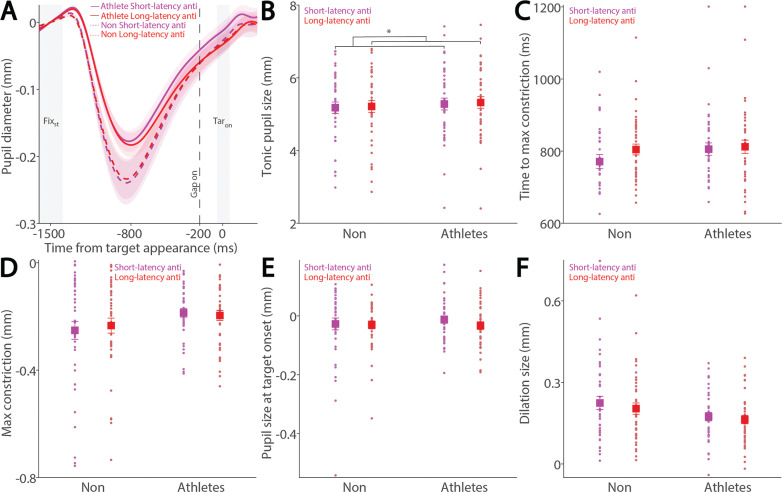
Pupil size for trials with faster and slower anti-saccades between athletes and non-athletes. (A) Pupil dynamics between the pro- and anti-saccade conditions in athletes and non-athletes. (B) Tonic pupil size in the FIX_st_ epoch is shown for different conditions between athletes and non-athletes. (C) Time to max constriction is shown for different conditions between athletes and non-athletes. (D) Max constriction magnitude is shown for different conditions between athletes and non-athletes. (E) Pupil size in the TAR_on_ epoch is shown for different conditions between athletes and non-athletes. (F) Dilation size is shown for different conditions between athletes and non-athletes. The shaded colored regions surrounding pupil dynamics curves represent the ± standard error range (across participants) for different conditions. The color-filled squares and error bars represent mean value ± standard error (across participants) for each condition, and the small circles represent mean value for each subject. Color dots represent each data point. The grey area represents the epoch selected for analyses. * indicates differences are statistically significant. Non: non-athletes.

To examine these effects in the anti-saccade condition, we analyzed correct anti-saccade trials based on SRT, including only regular-latency saccades and grouping them into faster and slower SRTs using a median split. Figure 4A shows pupil dynamics before target appearance in correct anti-saccade trials. Smaller tonic pupil sizes were observed in short-latency trials compared to long-latency trials (Fig. 4B, F_(1, 78)_ = 6.477, p = 0.013, ηp^2^ = 0.077), with no other significant effects (p > 0.6). No significant effects of saccade type, group, or interaction were found for time to maximum constriction (Fig. 4C, p > 0.09), max constriction magnitude (Fig. 4D, p > 0.06), or pupil size at target onset (Fig. 4E, p > 0.1). Notably, the interaction effect for max constriction magnitude approached significance (Fig. 4D, F_(1, 78)_ = 3.464, p = 0.067, ηp^2^ = 0.043). Larger pupil dilation was observed in short-latency trials compared to long-latency trials, although this effect did not reach significance (Fig. 4F, F_(1, 78)_ = 3.787, p = 0.055, ηp^2^ = 0.046), with no other significant effects (group: F_(1, 78)_ = 3.069, p = 0.084, ηp^2^ = 0.038; interaction: F_(1, 78)_ = 0.458, p = 0.501, ηp^2^ = 0.006).

**FIG. 5:**
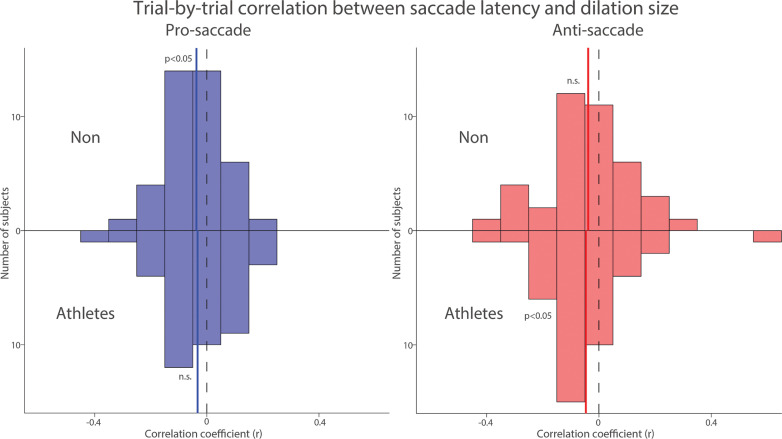
Trial-by-trial correlation between dilation size and saccade latency between athletes and non-athletes. Distribution of correlation coefficients (Fisher’s z-transformed) for the relationship between pupil dilation size and saccade reaction times across different conditions and groups. The vertical black dashed and colored solid lines represent the zero and median value of the correlation coefficient. n.s.: non-significant. Non: non-athletes.

To further examine the trial-by-trial relationship between pupil dilation and SRT, which was the primary focus of the study, we correlated pupil dilation magnitude with SRT for each subject in both groups. Figure 5 summarizes the distribution of correlation coefficients after applying Fisher’s z-transformation across all participants in both groups. Because previous studies have reported negative correlations between pupil dilation and SRTs [26, 27], we conducted one-tailed t-tests to assess this relationship. In both groups, negative correlations were observed in the pro- and anti-saccade conditions, such that larger pupil dilation tended to be associated with faster SRTs. However, statistical significance was reached only in the pro-saccade condition for the NON group (pro: t(39) = 2.295, p = 0.014, Cohen’s d = 0.363) and in the anti-saccade condition for the athlete group (anti: t(39) = 1.909, p = 0.032, d = 0.302). The other conditions showed non-significant trends in the same direction (p-values > .05). These results suggest a potential association between pupil metrics and SRTs, although the evidence was not consistently significant across all conditions. Overall, these results suggest that pupil metrics are associated with SRT in both groups.

### Larger pupil dilation in preparation for higher-velocity saccades

**FIG. 6:**
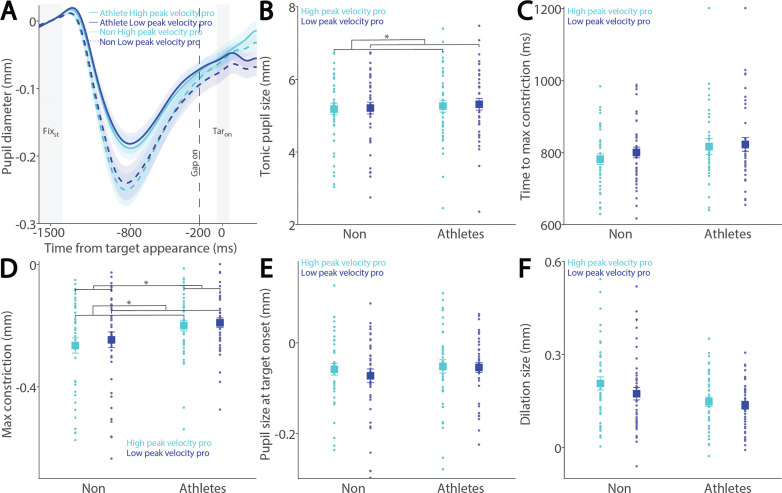
Pupil size for trials with higher and lower saccade peak velocities in pro-saccades between athletes and non-athletes. (A) Pupil dynamics between the pro- and anti-saccade conditions in athletes and non-athletes. (B) Tonic pupil size in the FIX_st_ epoch is shown for different conditions between athletes and non-athletes. (C) Time to max constriction is shown for different conditions between athletes and non-athletes. (D) Max constriction magnitude is shown for different conditions between athletes and non-athletes. (E) Pupil size in the TAR_on_ epoch is shown for different conditions between athletes and non-athletes. (F) Dilation size is shown for different conditions between athletes and non-athletes. The shaded colored regions surrounding pupil dynamics curves represent the ± standard error range (across participants) for different conditions. The color-filled squares and error bars represent mean value ± standard error (across participants) for each condition, and the small circles represent mean value for each subject. Color dots represent each data point. The grey area represents the epoch selected for analyses. * indicates differences are statistically significant. Non: non-athletes.

While the relationship between pupil dilation and SRT has been previously examined [26, 27], the influence of pupil dynamics on saccade metrics remains largely unexplored. To address this, we examined the relationship between pupil metrics and saccade peak velocity in both athletes and non-athletes. Figure 6A shows baseline-corrected pupil dynamics prior to target onset in correct pro-saccade trials, grouped by higher and lower saccade peak velocities using a median split. Smaller tonic pupil sizes were observed in trials with higher peak velocity compared to those with lower peak velocity (Fig. 6B, F_(1, 78)_ = 5.232, p = 0.025, ηp^2^ = 0.063), with no other significant effects (p > 0.5). No differences were found in time to maximum constriction (Fig. 6C, p > 0.1). In contrast, significant main effects of peak velocity and group were observed in max constriction magnitude (Fig. 6D; peak velocity: F_(1, 78)_ = 4.611, p = 0.035, ηp^2^ = 0.056; group: F_(1, 78)_ = 4.294, p = 0.042, ηp^2^ = 0.052), with greater constriction associated with higher velocities and no significant interaction (p > 0.4). Notably, the group effect on peak constriction magnitude was no longer significant after controlling for age (F_(1, 77)_ = 3.084, p = 0.083, ηp^2^ = 0.039). No differences were seen in pupil size at target onset (Fig. 6E, p > 0.2). More importantly, larger pupil dilation was observed in trials with higher peak velocities compared to those with lower velocities (Fig. 6F; peak velocity: F_(1, 78)_ = 14.499, p < 0.001, ηp^2^ = 0.157). Effects of group and the interaction did not reach statistical significance (group: F_(1, 78)_ = 3.938, p = 0.051, ηp^2^ = 0.048; interaction: F_(1, 78)_ = 3.517, p = 0.064, ηp^2^ = 0.043).

**FIG. 7:**
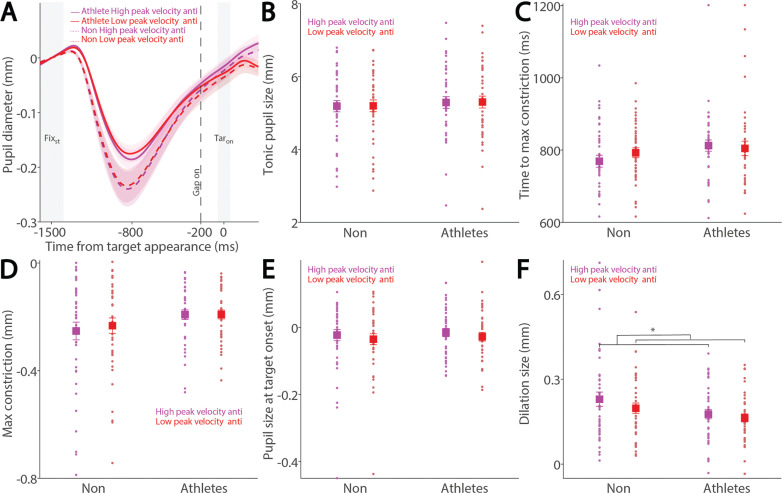
Pupil size for trials with higher and lower saccade peak velocities in anti-saccades between athletes and non-athletes. (A) Pupil dynamics between the pro- and anti-saccade conditions in athletes and non-athletes. (B) Tonic pupil size in the FIX_st_ epoch is shown for different conditions between athletes and non-athletes. (C) Time to max constriction is shown for different conditions between athletes and non-athletes. (D) Max constriction magnitude is shown for different conditions between athletes and non-athletes. (E) Pupil size in the TAR_on_ epoch is shown for different conditions between athletes and non-athletes. (F) Dilation size is shown for different conditions between athletes and non-athletes. The shaded colored regions surrounding pupil dynamics curves represent the ± standard error range (across participants) for different conditions. The color-filled squares and error bars represent mean value ± standard error (across participants) for each condition, and the small circles represent mean value for each subject. Color dots represent each data point. The grey area represents the epoch selected for analyses. * indicates differences are statistically significant. Non: non-athletes.

Similarly, Figure 7A shows baseline-corrected pupil dynamics prior to target onset in correct anti-saccade trials, including regular-latency saccades and grouped by higher and lower saccade peak velocities using a median split. No significant effects of peak velocity, group, or their interaction were found for tonic pupil size (Fig. 7B, p > 0.4) or time to max constriction (Fig. 7C, p > 0.1). Although greater pupil constriction was observed in trials with higher peak velocities, this effect was not significant (Fig. 7D; velocity: F_(1, 78)_ = 3.676, p = 0.059, ηp^2^ = 0.045), with no other significant effects (p > 0.1). No significant differences were found in pupil size at target onset (Fig. 7E, p > 0.1). More importantly, pupil dilation was significantly modulated by saccade peak velocity, with larger dilation associated with higher peak velocities (Fig. 7F; velocity: F_(1, 78)_ = 10.936, p = 0.001, ηp^2^ = 0.123), and no other significant effects (p > 0.1).

**FIG. 8:**
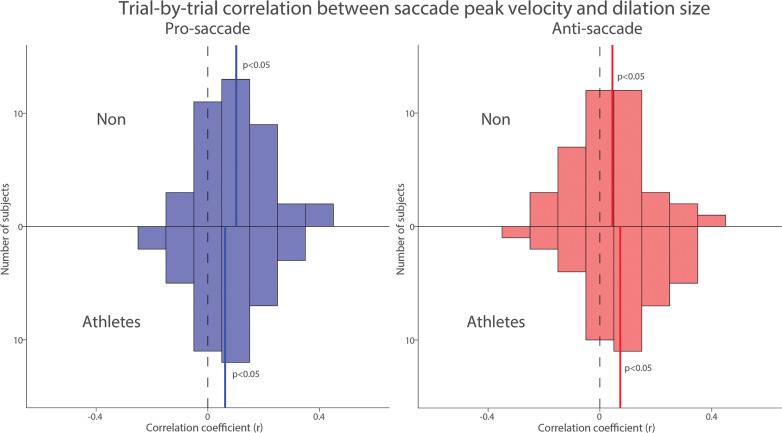
Trial-by-trial correlation between dilation size and saccade peak velocity between athletes and non-athletes. Distribution of correlation coefficients (Fisher’s z-transformed) for the relationship between pupil dilation size and saccade reaction times across different conditions and groups. The vertical black dashed and colored solid lines represent the zero and median value of the correlation coefficient. n.s.: non-significant. Non: non-athletes.

To examine trial-by-trial relationship between pupil dilation and saccade peak velocity, we correlated pupil dilation magnitude with saccade peak velocity for each subject in both groups. Figure 8 summarizes the distribution of correlation coefficients after applying Fisher’s z-transformation across all participants. Significant positive correlations between pupil dilation and saccade peak velocity were observed in both the pro- and anti-saccade conditions for both groups, indicating that larger pupil dilation was associated with higher saccade peak velocities (Fig. 8, NON: pro: t(39) = 5.353, p < 0.001, d = 0.846; anti: t(39) = 2.124, p = 0.040, d = 0.336; Athletes: pro: t(39) = 3.184, p = 0.003, d = 0.503; anti: t(39) = 3.176, p = 0.003, d = 0.502). Overall, these results suggest that pupil metrics before target appearance are associated with the speed of saccades generated in both groups.

### No correlation between pupil dilation and endpoint saccade deviation

We further examined the influence of pupil dynamics on saccade endpoint deviation by analyzing the relationship between pupil metrics and saccade endpoint deviation in both athletes and non-athletes. As shown in the supplementary figures, pupil metrics did not correlate with saccade endpoint deviation (Supplementary Fig. 2 and 3). Moreover, no trial-by-trial correlations between pupil dilation and saccade endpoint deviation were observed in either group (Supplementary Fig. 4). Together, these results suggest that there is no association between pupil metrics and saccade endpoint deviation in either condition or group.

## DISCUSSION

To understand preparatory set in athletes, we examined pupil dynamics during active preparation for pro-saccades or anti-saccades in the anti-saccade task, comparing athletes and non-athletes (NON). Larger pupil dilation was observed during pro-saccade preparation compared to anti-saccade preparation in both groups. More interestingly, athletes exhibited smaller pupil dilation compared to NON. Smaller tonic pupil sizes and larger pupil dilation were associated with faster SRTs. Moreover, trial-by-trial correlations between pupil dilation and SRTs were observed in both groups, although these effects were not consistently significant across all conditions. Additionally, pupil dilation was positively associated with saccade peak velocity, with larger pupil dilation correlating with higher peak velocities. In contrast, no associations between pupil dynamics and saccade endpoint deviation were observed. Overall, these results suggest that pupil responses prior to target appearance effectively reflect preparatory set activity related to saccade initiation in athletes. While reduced pupil dilation was observed in athletes, this dilation was effectively modulated by task preparation and correlated with saccade latency and peak velocity, possibly indicating more efficient preparation indexed by reduced pupil dilation in athletes compared to non-athletes.

### Pupillometry associated with saccade preparation in athletes

Athletes often exhibit cognitive advantages in behavioral tasks compared to NON [7, 8, 36–39], although contradictory findings have also been reported [43]. While conventional behavioral measures offer objective means to assess cognitive functions in athletes, pupillometry and eye movements can reveal subtle yet meaningful differences in underlying cognitive processes, thereby providing deeper insights into the cognitive foundations of athletic expertise. As a result, eye-tracking has become a widely adopted approach for examining cognition in athletes, or more broadly, the relationship between physical activity and cognitive function [44]. More specifically, pupil responses in the anti-saccade task have been applied in sports science research to examine executive functioning associated with postexercise or sport-related concussion [24, 45]. Consistent with previous research [23–28], we observed larger pupil dilation during anti-saccade preparation compared to pro-saccade preparation in both groups. More notably, although pupil dynamics prior to target appearance were qualitatively similar between athletes and NON, athletes exhibited reduced pupil constriction and smaller pupil dilation. Further analyses of the relationship between pupil responses and saccade behavior revealed that both pupil dilation and tonic pupil size were associated with SRTs. In line with prior findings [26, 27], trial-by-trial negative correlations between pupil dilation and SRTs were observed, although these effects were not consistently significant across all conditions. Smaller tonic pupil sizes were associated with faster SRTs, together suggesting that both phasic and tonic pupil measures could contribute to variability in preparatory efficiency.

We further explored whether pupil dynamics were linked not only to saccade latency but also to saccade metrics. Indeed, saccade peak velocity was positively correlated with pupil dilation, such that trials with larger pupil dilation exhibited higher peak velocities. In contrast, no associations were found between pupil responses and saccade endpoint deviation, indicating that pupil-linked preparatory processes primarily influence saccade timing and vigor, rather than endpoint accuracy. Taken together, our findings demonstrate that both phasic (pupil dilation) and tonic pupil responses modulate SRTs during the generation of pro- and anti-saccades. Moreover, pupil dilation was associated with both saccade latency and peak velocity, but not with saccade endpoint accuracy, suggesting a selective influence of pupillinked preparatory activity on motor readiness and movement dynamics. Importantly, although athletes showed reduced pupil dilation compared to NON, this response was still effectively modulated by task demands and remained predictive of saccade latency and velocity. These findings suggest that athletes may engage in more efficient motor preparation, using reduced pupil dilation to achieve effective task execution. Note that the interpretation that reduced pupil dilation in athletes reflects greater efficiency should be considered with caution, for several reasons. First, while this effect remained comparable in magnitude after controlling for age, it became marginally significant (p = 0.050). Moreover, pupil size is influenced by multiple factors beyond task-related preparatory activity [14, 15]. Although tonic pupil size was comparable between athletes and NON, suggesting similar arousal levels, differences in task familiarity or cognitive engagement due to repeated exposure to similar tasks may have contributed to the observed reduction in pupil dilation. Fatigue, although unlikely given the brief experimental duration, also cannot be entirely excluded. Therefore, while our findings are consistent with the idea of more efficient preparation in athletes, the current data do not support definitive causal inferences. Future studies incorporating physiological measures of arousal (e.g., heart rate variability), as well as assessments of task engagement and familiarity, will be important for distinguishing between these potential explanations. In summary, future research may explore whether pupil dilation can serve as a physiological index of individual athletic ability, offering a potential objective marker of motor planning load and preparatory efficiency in athletes. Nevertheless, our findings extend prior work on cognitive–motor integration by demonstrating that pupil-linked preparatory signals, modulated by executive control, are associated with both saccade latency and saccade dynamics, consistent with literature linking executive function to motor components in athletic, or physical activity more generally [7, 8, 46].

### Neural mechanisms underlying pupil-linked preparatory effects in athletes

Distinct neural networks are involved in the active preparation for pro- and anti-saccades [3–6]. Unlike the automatic pro-saccade response that is directly triggered by target appearance, successful execution of an anti-saccade requires top-down inhibitory control to suppress the automatic response and volitionally generate a goaldirected saccade in the opposite direction. The SC and the FEF are anatomically and physiologically connected [47], and both structures are actively engaged during saccade preparation. Specifically, studies from both humans and monkeys have shown greater neural activity in the SC and FEF during anti-saccade preparation compared to pro-saccade preparation, and this preparatory activity has been shown to negatively correlate with SRTs [3, 9–12], suggesting that higher neural activation facilitates faster saccade initiation. A similar pattern has been reported in pupil dilation, with larger pupil dilation observed during anti-saccade compared to pro-saccade preparation, and trial-by-trial pupil dilation negatively correlates with SRTs [23–28]. These findings suggest that pupil dilation reflects task preparation and is associated with oculomotor network activation. Consistent with this interpretation, pupil dilation can be evoked by microstimulation of the SC or FEF in behaving monkeys [20–22]. Importantly, pupil size is modulated not only by SC-mediated circuits but also by LC-mediated circuits [14, 15]. The tonic mode of LC activity is associated with tonic (pre-stimulus) pupil size [17], indicating that pupil responses may reflect neural activity associated with both SC and LC.

In our study, we found larger pupil dilation during anti-saccade compared to pro-saccade preparation in both groups, likely reflecting oculomotor circuit activation associated with task preparation. More intriguingly, athletes exhibited reduced pupil dilation compared to NON, possibly indicating lower neural activation during preparation in athletes. This reduced activation, while still responsive to task demands, may suggest more efficient neural recruitment in athletes during motor planning. Moreover, pupil dilation was not only associated with SRTs but also with saccade peak velocity, thereby linking pupil responses to the movement dynamics of saccades. These results align with previous findings that SC activity is related to saccade velocity [33, 34]. Notably, no correlation was observed between pupil dilation and saccade endpoint deviation, supporting the idea that the SC predominantly contributes to the initial acceleration phase of saccades rather than endpoint accuracy [48]. Additionally, tonic pupil size modulated SRTs, suggesting a possible role of the LC in task control. Given that the SC receives both anatomical and functional input from the LC [49], it is plausible that LC-mediated modulation of tonic pupil size influences saccade behavior via its projections to the SC. Together, these findings support the idea that the SC serves as a hub that integrates preparatory signals from the FEF and the arousal signals from the LC to coordinate saccade latency and dynamics. Both phasic pupil dilation and tonic pupil size appear to reflect complementary aspects of this integration, contributing to the temporal and dynamic control of eye movements during goal-directed saccade generation.

### Limitations and future directions

The current study included only athletes from open-skill sports, as they often exhibit cognitive advantages in lab-based behavioral tasks compared to non-athletes [36–39]. To gain a more comprehensive understanding of the relationship between pupil dynamics and preparatory processes in athletes, it is important for future research to include athletes from closed-skill sports as well. Moreover, studies examining athletes across different sports have reported differences in saccade behavior [50]. Future investigations should systematically compare athletes across a range of sport types to extend our understanding of preparatory set and its modulation across different athletic domains. Moreover, although our sample size (40 athletes and 40 controls) was based on previous studies with comparable designs and measurements, several effects in the present study only approached significance, raising concerns about the underlying effect sizes. To address this, a post hoc sensitivity analysis was conducted using G*Power [51]. With α = .05 and power set at 80%, our sample size was sufficient to detect between-group effects with a minimum effect size of partial η^2^ = 0.088 (Cohen’s f = 0.32), and a within-subjects or interaction effect with a minimum effect size of partial η^2^ = 0.046 (Cohen’s f = 0.22), which corresponds to a medium and small effect, respectively. This suggests that the study was adequately powered to detect moderate effects but may have lacked the sensitivity to identify smaller, potentially meaningful effects reliably. Future research would benefit from conducting a formal a priori power analysis to optimize sample size and ensure sufficient statistical power to detect subtle but potentially meaningful effects. We used an interleaved pro- and anti-saccade task, a wellestablished paradigm commonly used to examine preparatory set and its underlying neural mechanisms [3–6]. However, this lab-based task differs substantially from the demands required for real-world sports environments. Research incorporating ecologically valid, dynamic sports tasks that more closely resemble real-life conditions is thus essential to validate and generalize the present findings in applied sport settings.

## CONCLUSIONS

Pupillometry offers a simple yet powerful method to study both lowlevel sensory processing and high-level cognitive functions. In this study, we demonstrated differences in preparatory processes between athletes and non-athletes, with both pre-stimulus arousal signals and task-related preparatory signals contributing to task performance. These results align with recent research [52], which highlighted the pupil light reflex as an objective physiological measure for assessing fatigue levels in athletes. Collectively, these findings underscore the value of pupil size as a physiology-based, objective, and informative index for examining a range of cognitive, arousal, and motor processes in sports science research.

## Supplementary Material

Pupil dynamics reveal preparatory processes in the generation of pro-saccades and anti-saccades in open skill sports athletes

## Data Availability

Data are available from the corresponding author upon reasonable request following publication.
