# Supplementary material for: Pupil dynamics reveal preparatory processes in the generation of pro-saccades and anti-saccades in open skill sports athletes
Source: Biol Sport. 2025 Aug 5;43:77–94. doi: 10.5114/biolsport.2026.153308 (PMC12884883; doi:10.5114/biolsport.2026.153308)
Supplement: Pupil dynamics reveal preparatory processes in the generation of pro-saccades and anti-saccades in open skill sports athletes [file JBS-43-56513-s1.pdf]

SUPPLEMENTARY MATERIAL

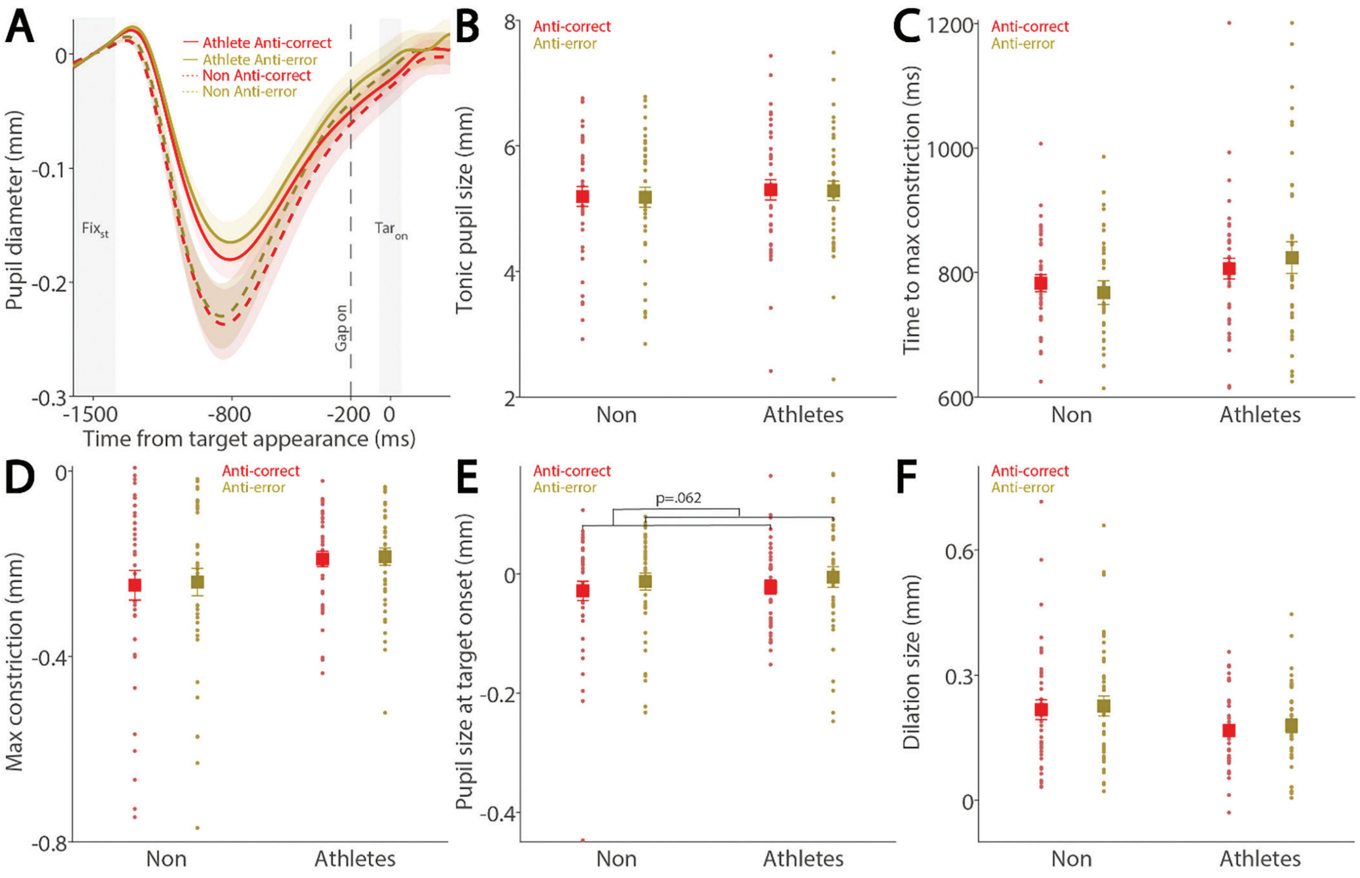

SUPPLEMENTARY FIGURE 1. Pupil size modulation on erroneous anti-saccade trials between athletes and non-athletes. (A) Pupil dynamics between the correct and erroneous anti-saccade trials in athletes and non-athletes. (B) Tonic pupil size in the FIX_{st} epoch shown for different conditions between athletes and non-athletes. (C) Time to max constriction shown for different conditions between athletes and non-athletes. (D) Max constriction magnitude shown for different conditions between athletes and non-athletes. (E) Pupil size in the TAR_{on} epoch shown for different conditions between athletes and non-athletes. (F) Dilation size shown for different conditions between athletes and non-athletes. The shaded colored regions surrounding pupil dynamics curves represent the ± standard error range (across participants) for different conditions. The color-filled squares and error-bars represent mean value ± standard error (across participants) for each condition, and the small circles represent mean value for each subject. Color dots represent each data point. The gray area represents the epoch selected for analyses. * indicates differences are statistically significant. Non: non-athletes.

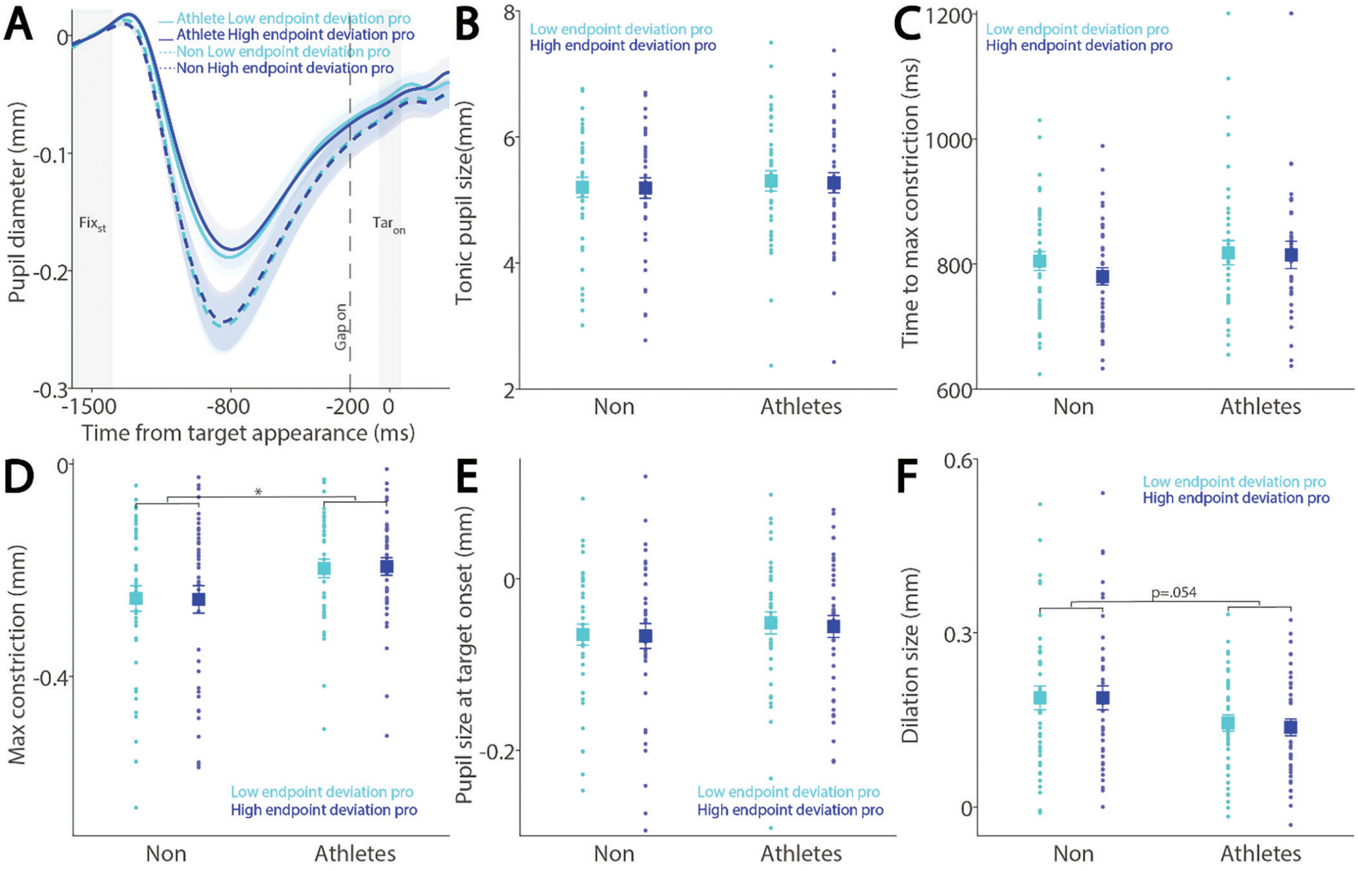

SUPPLEMENTARY FIGURE 2. Pupil size for trials with lower and higher saccade endpoint deviation in pro-saccades between athletes and non-athletes. (A) Pupil dynamics between the pro- and anti-saccade condition in athletes and non-athletes. (B) Tonic pupil size in the FIX_{st} epoch shown for different conditions between athletes and non-athletes. (C) Time to max constriction shown for different conditions between athletes and non-athletes. (D) Max constriction magnitude shown for different conditions between athletes and non-athletes. (E) Pupil size in the TAR_{on} epoch shown for different conditions between athletes and non-athletes. (F) Dilation size shown for different conditions between athletes and non-athletes. The shaded colored regions surrounding pupil dynamics curves represent the ± standard error range (across participants) for different conditions. The color-filled squares and error-bars represent mean value ± standard error (across participants) for each condition, and the small circles represent mean value for each subject. Color dots represent each data point. The gray area represents the epoch selected for analyses. * indicates differences are statistically significant. Non: non-athletes.

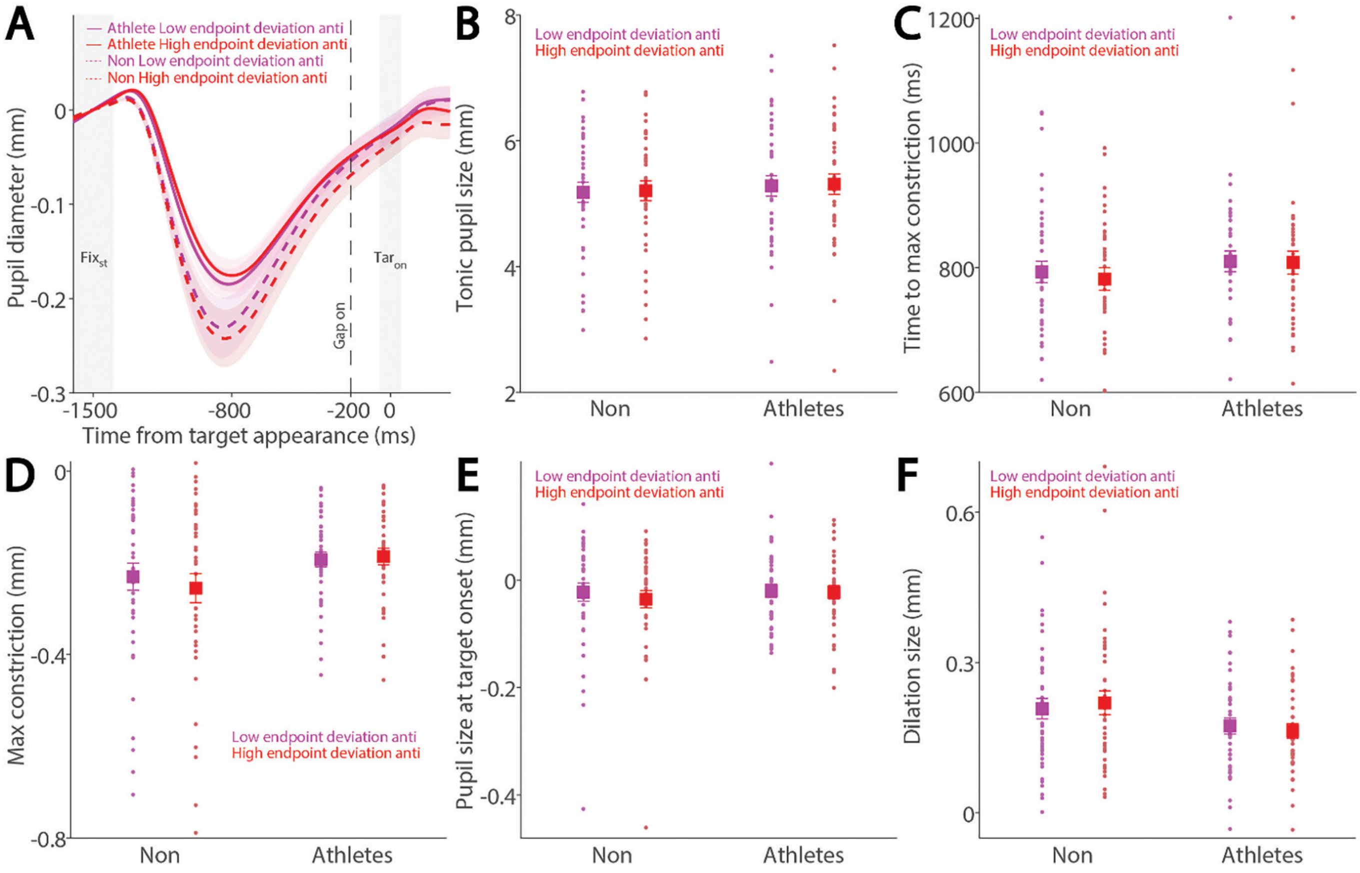

SUPPLEMENTARY FIGURE 3. Pupil size for trials with lower and higher saccade endpoint deviation in anti-saccades between athletes and non-athletes. (A) Pupil dynamics between the pro- and anti-saccade condition in athletes and non-athletes. (B) Tonic pupil size in the FIX_{st} epoch shown for different conditions between athletes and non-athletes. (C) Time to max constriction shown for different conditions between athletes and non-athletes. (D) Max constriction magnitude shown for different conditions between athletes and non-athletes. (E) Pupil size in the TAR_{on} epoch shown for different conditions between athletes and non-athletes. (F) Dilation size shown for different conditions between athletes and non-athletes. The shaded colored regions surrounding pupil dynamics curves represent the ± standard error range (across participants) for different conditions. The color-filled squares and error-bars represent mean value ± standard error (across participants) for each condition, and the small circles represent mean value for each subject. Color dots represent each data point. The gray area represents the epoch selected for analyses. * indicates differences are statistically significant. Non: non-athletes.

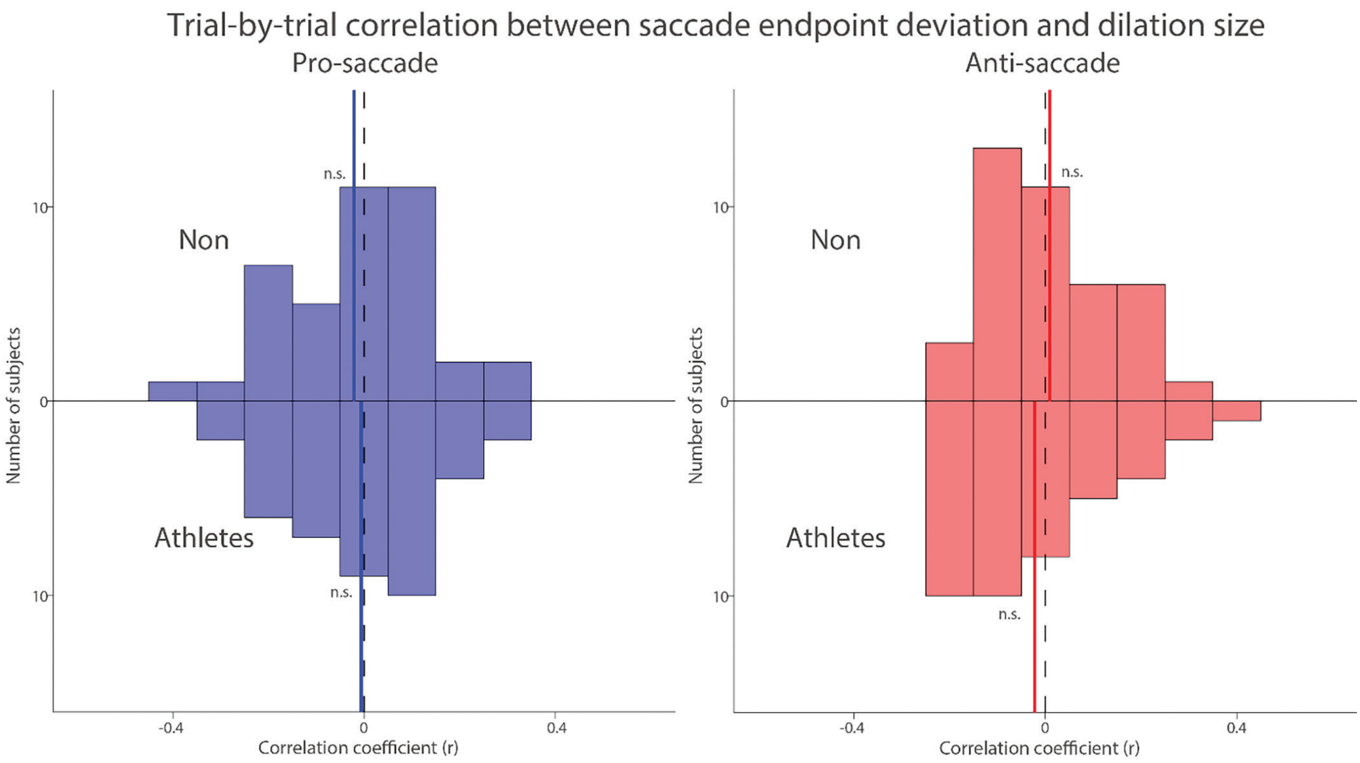

SUPPLEMENTARY FIGURE 4. Trial-by-trial correlation between dilation size and saccade endpoint deviation between athletes and non-athletes. Distribution of correlation coefficients (Fisher's z-transformed) for the relationship between pupil dilation size and saccade reaction times across different conditions and groups. The vertical black dashed and colored solid line represent a zero and median value of correlation coefficient. n.s.: non-significant. Non: non-athletes.

Articles published in the Biology of Sport are licensed under an open access Creative Commons CC BY 4.0 license.
